# The Extent of Transmission of Novel Coronavirus in Wuhan, China, 2020

**DOI:** 10.3390/jcm9020330

**Published:** 2020-01-24

**Authors:** Hiroshi Nishiura, Sung-mok Jung, Natalie M. Linton, Ryo Kinoshita, Yichi Yang, Katsuma Hayashi, Tetsuro Kobayashi, Baoyin Yuan, Andrei R. Akhmetzhanov

**Affiliations:** 1 Graduate School of Medicine, Hokkaido University, Kita 15 Jo Nishi 7 Chome, Kita-ku, Sapporo-shi, Hokkaido 060-8638, Japan; seductmd@med.hokudai.ac.jp (S.-m.J.); nlinton@gmail.com (N.M.L.); kinoshitaryo@gmail.com (R.K.); lukeyang1993@eis.hokudai.ac.jp (Y.Y.); katsuma5miffy@gmail.com (K.H.); tootsieroll2910@gmail.com (T.K.); baoyinyuan@gmail.com (B.Y.); akhmetzhanov@gmail.com (A.R.A.); 2 CREST, Japan Science and Technology Agency, Honcho 4-1-8, Kawaguchi, Saitama 332-0012, Japan

**Keywords:** epidemiology, foreigner, travel, migration, importation, emerging infectious diseases

## Abstract

A cluster of pneumonia cases linked to a novel coronavirus (2019-nCoV) was reported by China in late December 2019. Reported case incidence has now reached the hundreds, but this is likely an underestimate. As of 24 January 2020, with reports of thirteen exportation events, we estimate the cumulative incidence in China at 5502 cases (95% confidence interval: 3027, 9057). The most plausible number of infections is in the order of thousands, rather than hundreds, and there is a strong indication that untraced exposures other than the one in the epidemiologically linked seafood market in Wuhan have occurred.

## 1. Introduction

Since the announcement of a cluster of pneumonia cases of unknown etiology in Wuhan, Hubei Province, China, was made on 31 December 2019, many rapid virological, clinical, and epidemiological research responses have taken place [1,2]. The causative agent of the pneumonia is suggested to be a novel coronavirus (2019-nCoV) of the same lineage (but genetically distinct) from the coronavirus causing severe acute respiratory syndrome (SARS) [1]. Cases in the initial cluster reported a common exposure—a seafood market in Wuhan where wild animals were served at a restaurant—indicating that a point-source zoonotic (animal-to-human) route was likely the main mode of transmission for those cases [2]. 

Although early reports from Wuhan [3] stated that (i) there were only tens of cases in the cluster and (ii) no human-to-human transmission was directly observed, the scientific community was alert to the possibility that the novel coronavirus would spread to other geographic locations—including other countries—via direct human-to-human transmission. In early January, the outbreak began to escalate rapidly with hundreds of cases now confirmed along with the presence of a few household clusters [4,5,6,7]. 

As of 24 January 2020, the cumulative incidence in China is 830 cases, of which 549 cases were diagnosed in Hubei, 26 in Beijing, 20 in Shanghai, and 53 in Guangdong. Additionally, twenty-six deaths have been linked to the outbreak [6,8], and thirteen cases were exported to Japan, Singapore, South Korea, Taiwan, Thailand, Vietnam and the United States as of 22 January 2020. Considering that enhanced surveillance has been underway in these importing countries, case ascertainment has been perhaps better in exported case data.

Using a spatial back-calculation method and analyzing exported cases, we estimate the cumulative incidence of 2019-nCoV cases in China in real time, allowing us to update and discuss the extent of transmission at the source.

## 2. Exported Cases

Table 1 shows the incidence of exported cases by date of hospitalization and report. Due to the initial difficulty of diagnosis in the absence of established primer for polymerase chain reaction testing, the time lag between hospitalization and reporting was longer for early cases compared with that of more recent cases. Among the seven locations reporting importation, the total volume of inbound passengers from China was *m* = 63.1 million per year in 2017 [9], of which 100*q* = 2.1% were from Wuhan [10], a home of *n* = 19.0 million people as the catchment population of Wuhan airport. Two other locations with confirmed cases, i.e., Macau and Hong Kong, were excluded from the analysis, because it is commutable by land transporation and the first case in Hong Kong was indeed not via airtravel. As we already know from elsewhere [11,12,13], given the observed cumulative count of *c* exported cases, we have a balance equation of the cumulative risk of infection:

(1) $$\hat{p}=c\frac{365}{mqT},$$

where *T* is the sum of incubation and infectious periods, and here is assumed to be 3.2 and 9.3 days [14], respectively, assuming that these periods are similar to those of other coronaviruses, and thus, *T* = 12.5 days. The estimated incidence in China is then given by $\hat{p}n$. With an ad-hoc assumption that the data are generated following the binomial sampling process among travelers from Wuhan, the cumulative incidence is then estimated using a maximum likelihood method.

Table 1 also shows the estimated incidence in China. The first exportation event in Thailand suggests 423 cases with the upper confidence limit of 1863 cases. The estimated cumulative incidence has grown as additional cases have been reported. As of 24 January 2020, with reports of thirteen exportation events, the cumulative incidence in China is estimated at 5502 cases (95% confidence interval: 3027, 9057). 

Our latest estimate is comparable to a preliminary report posted by a research group at Imperial College London (ICL) on their own homepage on 22 January 2020 [26] that estimated the incidence based on three importation events at 4000 cases (95% CI: 1000, 9700). Possible reasons for the slight difference include (i) the number of travelers in the previous study was derived from airline passenger data [27] and (ii) the assumed length of *T* was different. Two other estimates have also been published: a preliminary study by a Northeastern University group estimated 1250 cases (95% CI: 350, 3000) as of 17 January 2020 [28] and a University of Hong Kong group estimated 1343 cases (95% CI: 547, 3446) as of 17 January 2020 [29]. The former study from the United States assumes that the catchment area population is 10 million (we use 11.1 million).

## 3. The Way Forward

The number of reported 2019-nCoV infections continues to grow as surveillance and detection methods improve. Our estimate and others [26,28,29] agree that the actual number of cases is likely in the order of thousands, rather than hundreds, and there is a strong indication that untraced exposures other than that of the originally linked seafood market in Wuhan have occurred. Such exposures are expected to include human-to-human transmission, but the levels of transmissibility have yet to be quantified. It is still plausible that a substantial number of human infections arose from animal-to-human exposures, such as was the case during the first outbreak of highly pathogenic influenza (H7N9) in China, 2013, and the human-to-human transmissibility has yet to be quantified in an explicit manner. 

Despite initially restricting what information on the outbreak was shared publicly, the Chinese government has begun to respectfully provide updates on the situation on a daily basis. This encourages the real-time release of information by means of regularly updated situation reports, including epidemiological information with dates of exposure, illness onset, and hospitalization among cases.

For researchers to be able to contribute to control efforts by improving situation awareness via an explicit risk assessment, it is crucial that detailed epidemiological data are posted to a public domain in real-time. Such datasets should include not only a deidentified line list of cases but also updates on the infection status of traced contacts. Information on exposure period and illness onset can assist with the estimation of important natural history parameters such as the incubation period. It is critical for the public health community and the public at large to understand more about the process of case ascertainment, including the current case definition and reporting system mechanisms.

## Figures and Tables

**Table 1:** Exportation events and estimated incidence in China.

Importing location	Date of Hospitalization	Date of Report	Cumulative Count	Estimated Incidence in China (95% CI)
Thailand	8 January	14 January	1	423 (NC, 1863)
Japan	10 January	16 January	2	846 (141, 2614)
Thailand	13 January	17 January	3	1270 (316, 3292)
South Korea	19 January	20 January	4	1693 (526, 3933)
Taiwan	19 January	21 January	5	2116 (759, 4548)
United States	19 January	21 January	6	2539 (1009, 5145)
Thailand	19 January	22 January	7	2962 (1273, 5729)
Thailand	NA	22 January	8	3385 (1547, 6302)
Singapore	22 January	23 January	9	3809 (1830, 6866)
Vietnam	22 January	23 January	10	4231 (2121, 7423)
Vietnam	22 January	23 January	11	4655 (2418, 7973)
Japan	22 January	24 January	12	5078 (2720, 8517)
South Korea	22 January	24 January	13	5502 (3027, 9057)

CI, confidence interval (the 95% CI was derived from bootstrapping); NC, not calculable; NA, not available. The estimated incidence is updated as a function of the date of the report [15,16,17,18,19,20,21,22,23,24,25].
